# Comparing surgical outcomes of anterior capsular release vs circumferential release for persistent capsular stiffness

**DOI:** 10.1177/17585732221092016

**Published:** 2022-04-05

**Authors:** Safiya Erdogan, Seaher Sakha, Ajaykumar Shanmugaraj, Carlos Prada, Rachel M Frank, Timothy Leroux, Moin Khan

**Affiliations:** 1 Faculty of Health Sciences, 3710 McMaster University, Hamilton, Ontario, Canada; 2 Faculty of Life Sciences, 3710 McMaster University, Hamilton, Ontario, Canada; 3 Division of Orthopaedics, 3710 McMaster University, Hamilton, Ontario, Canada; 4 12225 University of Colorado, Boulder, CO, USA; 5 Division of Orthopaedic Surgery, University of Toronto, Toronto, Ontario, Canada; 6 Department of Health Research Methods, Evidence, and Impact, 3710 McMaster University, Hamilton, Ontario, Canada

**Keywords:** adhesive capsulitis, 360°, anterior release, frozen shoulder, arthroscopy

## Abstract

**Purpose:**

To consolidate the existing literature evaluating anterior capsular release and circumferential capsular release in the treatment of adhesive capsulitis (AC) of the shoulder.

**Methods:**

The electronic databases PUBMED, EMBASE, MEDLINE and CENTRAL (Cochrane Central Register of Controlled Trials) were searched from data inception to October 8, 2020. Data are presented descriptively where appropriate. A meta-analysis was conducted for patient-reported outcomes.

**Results:**

Overall, there were forty-six articles included. The majority of patients underwent circumferential release compared to anterior release (80.1% vs. 19.9%). Concomitant Manipulation Under Anesthesia (MUA) was employed in 25 studies, with a higher occurrence in the anterior compared to the circumferential release group (70% vs 60%). Both groups experienced significant improvements postoperatively in range of motion (ROM) and patient-reported outcomes. Complication rates were low for both anterior release (0.67%) and 360° release (0.44%).

**Conclusion:**

Both anterior and circumferential release are effective techniques for treating AC with low complication rates. Future studies should improve documentation of patient demographics, surgical techniques and outcomes to determine an individualized treatment protocol for patients.

**Level of evidence:**

Level IV, Systematic Review of Level I–IV studies.

## Introduction

Adhesive capsulitis (AC) is characterized by progressive loss of active and passive motion of the shoulder, leading to stiffness and pain.^1,2^ This condition is known to affect 2–5% of the general population and a higher proportion of patients with diabetes (10–36%).^3,4^ In addition to diabetes, risk factors for developing AC include trauma, thyroid disease and female sex.^1^

In cases where conservative treatment fails, surgical management is considered. This includes manipulation under anesthesia (MUA) often in combination with arthroscopic capsular release. MUA alone results in improvements in shoulder motion and function between 6 to 9 months from the onset of symptoms but places a patient at risk of injury to soft tissues or fracture,^5^ MUA is thus accompanied generally with arthroscopic capsular release to reduce the risk of iatrogenic injury with the literature supporting significant improvement seen in long-term outcomes with respect to clinical outcome scores and range of motion (ROM).^6^ Often, the anterior structures of the capsule are released arthroscopically. However, an extended release of the capsule, also known as a circumferential release (360°) can also be used.^7^ Employing the circumferential release technique avoids the need for manipulation in the majority of cases, which is routinely carried out during anterior capsular release, and may presents a potential for complication.^8^ These include rotator cuff tear, humeral and glenoid fractures and nerve damage.^9,10^ However, there is concern that release of the entire capsule could be associated with instability and potential axillary nerve damage during release of the inferior capsule.^8,11^ Outcomes of both procedures have been demonstrated to be effective in improving ROM with low revision and complication rates.^12,13^

It remains controversial as to whether an extended capsular release results in improved outcomes over isolated anterior release.^10^ The aim of this systematic review is to evaluate available literature to determine if there is any benefit to 360° capsular release over anterior capsular release with respect to ROM, functional outcomes as well as risk of complications.

## Methods

This systematic review was conducted according to the methods outlined in the Cochrane Handbook for Systematic Reviews and reported following the Preferred Reporting for Systematic Reviews and Meta-Analyses (PRISMA) guidelines.^14,15^

### Search strategy

PUBMED, EMBASE, MEDLINE and CENTRAL were searched on October 8, 2020 for literature on anterior and 360° capsular arthroscopic release for AC. The search terms included “shoulder”, “AC”, “anterior capsular release”, “360° capsular release” and similar phrases (Appendix I). MeSH and EMTREE terms were used in various combinations and supplemented with free text to increase search sensitivity. The search terms were entered onto Google Scholar and ClinicalTrials.gov, to ensure that relevant articles were not excluded. A hand search of the references of included articles was performed to ensure no potentially eligible articles were missed.

### Eligibility criteria

Inclusion criteria were: (1) anterior capsular release or 360° capsular release; (2) AC or frozen shoulder; (3) at least one outcome reported and stratified for population of interest; (4) human studies; and (5) English language. The exclusion criteria were: (1) shoulder condition not involving AC (e.g. rotator cuff tear); (2) neither anterior capsular release or 360° capsular release (e.g. release of anterior and posterior capsules only) as the surgical intervention; (3) review articles; (4) non-surgical treatment studies (e.g. conservative treatment, technique articles without outcomes, etc.); (5) cadaver/non-human/biomechanical studies; and (6) case reports.

### Study screening

The study screening was performed in duplicate by two independent reviewers (S.E., S.S.). Discrepancies that occurred at the title and abstract stage were resolved through discussion and consensus. If consensus could not be reached a senior reviewer was consulted when necessary (A.S.).

### Quality assessment

Using the Journal of Bone & Joint Surgery (JBJS) classification system for literature in the field of orthopaedics, the two reviewers determined the level of evidence (I to IV) for each study independently and in duplicate. The methodological quality of non-randomized comparative studies was evaluated using the methodological index for nonrandomized studies (MINORS). A score of 0, 1 or 2 is given for each of the 12 items on the MINORS checklist with a score of up to 16 for non-comparative studies and 24 for comparative studies. Methodological quality was categorized *a priori* as follows: a score of 0–8 or 0–12 was considered poor quality, 9–12 or 13–18 was considered fair quality, and 13–16 or 19–24 was considered excellent quality, for non-comparative and comparative studies, respectively. The Cochrane Risk of Bias tool was used to evaluate the quality of randomized trials. The Cochrane Risk of bias tool evaluates studies in 7 domains (i.e. random sequence generation, allocation concealment, selective reporting, blinding of participants and personnel, blinding of outcome assessment, outcome data, and other biases) as having high, unclear, or low risk of bias.

### Data abstraction

Two reviewers (S.E., S.S.) independently abstracted relevant data from included articles and recorded the data onto an Excel Spreadsheet designed *a priori*. Demographic data included author, year of publication, sample size, level of evidence and patient demographics (e.g. gender, age, etc.). Information regarding surgical techniques, rehabilitation protocols, post-operative outcomes and complications was documented.

### Statistical analysis

For relevant measures, descriptive statistics such as mean, range and measures of variance (e.g. standard deviations, 95% confidence intervals [CI]) are presented where applicable. A standard deviation was approximated using the range (i.e. [maximum-minimum]/4) for studies that did not report this for outcome measures.^16,17^ The intraclass correlation coefficient (ICC) was used to evaluate inter-reviewer agreement for assessing study quality. At all stages of screening, a kappa (κ) statistic was used to evaluate inter-reviewer agreement. The agreement was categorized *a priori* as follows: ICC/κ of 0.81 to 0.99 was considered an almost perfect agreement; ICC/κ of 0.61 to 0.80 as substantial agreement; ICC/κ of 0.41 to 0.60 as moderate agreement; 0.21 to 0.40 fair agreement and an ICC/κ value of 0.20 or less was evaluated as slight agreement. Review Manager 5.4 (The Cochrane Collaboration, 2020) was used to perform a meta-analysis. Continuous data were presented as mean differences (MD) with a 95% confidence interval (CI). Dichotomous data were presented as odds ratios (OR) with a 95% CI. The χ2 and I^2^ statistics were used to measure the heterogeneity of results within the included studies. For the χ2 test, a *p* < 0.05 was considered significant. The I^2^ test was categorized as follows: 0.0%–24.9% indicating no heterogeneity, 25.0%–49.9% indicating low heterogeneity; 50.0%–74.9% indicating moderate heterogeneity; 75.0%–100.0% indicating high heterogeneity. Furthermore, the random-effects model was used due to expected clinical heterogeneity.

## Results

### Study characteristics

**Figure 1.**
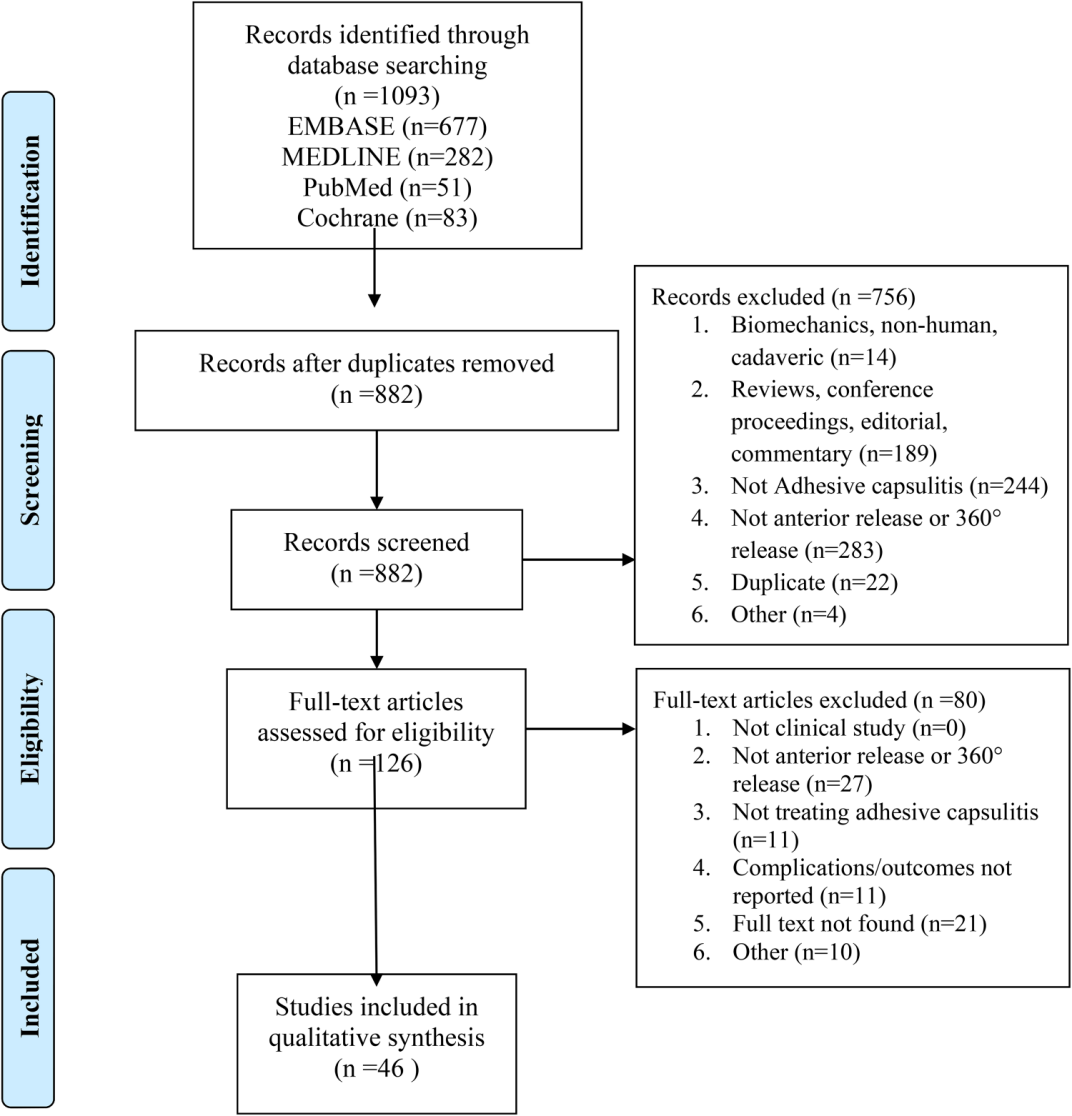
PRISMA flow diagram.

A total of 1093 studies were retrieved from the initial search and following title and abstract screening, 126 studies underwent full-text review. Of these studies, 46 studies met inclusion criteria (Figure 1). Studies were published between 1989 and 2020, most commonly from the USA (15.2%, n = 7), the United Kingdom (13.0%, n = 6), Korea (10.9%, n = 5) and Japan (10.9%, n = 5).

Within the 46 studies, a total of 2261 patients were included with a mean age of 54.0 (SD 5.0) and a mean follow-up of 29.3 months (SD 19.0). Within the overall study group, 80.1% (n = 1810) patients underwent circumferential release in 30/46 studies with a mean age of 54.21 (SD 4.4) and mean follow-up of 30.9 months (SD 17.7). In 10/46 studies, there were 19.9% (n = 451) patients who underwent an anterior/selective release with a mean age of 52.5 (SD 6.1) and a mean follow-up of 32.3 months (SD 23.0) (Table 1).

**Table 1.** Study characteristics.

			Patient characteristics			Treatment arm (# patients)		Follow-up			Rehabilitation		Study characteristics	
Study	Year	Country	Male sex, %		Age, yr	Surgical		Mean (months)	Loss to follow-up	Outcome measures	Immobilization (# weeks)	Immediate vs delayed motion	Type of Study	Level of Evidence
Bennett et al.	2000	USA	22.6	16.1	60.2	Anterior (18)	circumferential (13)	18	Not reported	ROM, Patient Satisfaction, Constant score, ASES, VAS	Not reported	Not reported	Prospective Cohort	3
Chen et al.	2010	China	Not reported	Not reported	56	Anterior + MUA (42)	Circumferential + MUA (32)	28	4	VAS 2wks, 4wks, 2mo, 3 mo, 6mo, final, ROM	4	Immediate (Day 1)	RCT	1
Kim et al.	2014	Korea	13.0	15.6	56.4	Anterior (37)	Circumferential (38)	18.4	2	VAS, ASES, ROM, SST 3mo, 6mo, 12mo, final	Not reported	Immediate (Day 1)	RCT	1
Moon et al.	2015	Korea	17.1	8.6	56	Anterior (19)	Circumferential (16)	8.9	Not reported	VAS, Constant, ROM postop, 1mo, 3mo, 6mo	Not reported	Immediate (Day 1)	Prospective Cohort	2
Ogilvie et al.	1995	Canada	70		Not reported	Anterior + MUA		Not reported	Not reported	Function scores, ROM, Pain scores	Not reported	Immediate (Day 0)	Prospective Cohort	2
Ozaki et al.	1989	Japan	58.9		53.5	Anterior + MUA		81.6	Not reported	Mean # vessels, vessel density	Not reported	Day 3	Prospective Cohort	2
Tsai et al.	2017	Taiwan	42.3		51.2	Anterior + MUA		28.2	Not reported	VAS, Constant, ASES, UCLA, ROM, Strength	Not reported	Immediate (Day 1)	Case Series	4
Warner et al.	1996	USA	47.8		Not reported	Anterior + MUA		39	Not reported	Constant score, ROM	Not reported	Immediate (Day 1)	Prospective Cohort	2
Omari et al.	2001	United Kingdom	52.0		52.6	Anterior		Not reported	Not reported	VAS, ROM assessment, ASES,	Not reported	Immediate (Day 1)	Prospective Cohort	2
Barnes et al.	2016	Australia	33.8		56	Circumferential + MUA		Not reported	Not reported	ROM	Not reported	Immediate (Day 1)	Case Series	4
Baums et al.	2007	Germany	46.7		50	Circumferential + MUA		36	Not reported	ASES, SST, VAS, ROM 6wks, 3mo, 6mo, 12mo, 36mo	Not reported	Immediate (day 0)	Prospective Cohort	2
Cho et al.	2016	Korea	27.0		55.6	Circumferential + MUA		48.4	8	VAS, UCLA, ASES, ROM 3mo, 6mo, 12mo, final	Not reported	Immediate (Day 0)	Retrospective Cohort	3
Cvetanovich et al.	2018	USA	30.3		54.8	Circumferential		44.4	6	ROM	Not reported	Immediate (Day 1)	Retrospective Case Series	4
Dattani et al.	2013	United Kingdom	40.0		54.3	Circumferential		Not reported	Not reported	ROM 6mo	Not reported	Immediate (Day 1)	Prospective Cohort	2
De Carli et al.	2011	Italy	47.8		57	Circumferential + MUA		Not reported	Not reported	UCLA, ASES, Constant, SST 3wks, 6wks, 12 wks, 6mo, 12mo, patient satisfaction,	2 weeks max	Immediate (Day 1)	Prospective Cohort	2
Diwan et al.	2005	Australia	Not reported		Not reported	Anterior + MUA (18)	Circumferential + MUA (22)	Not reported	Not reported	ROM intraoperatively	Not reported	Immediate (Day 0)	Case Control Cohort	3
Ebrahimzadeh et al.	2014	Iran	35.0		50.8	Circumferential + MUA		47	Not reported	VAS, Constant, DASH, ROWE, ROM	Not reported	Immediate (Day 0)	Prospective Cohort	2
Fernandes et al.	2013	Brazil	22.2		53.6	Circumferential		Not reported	Not reported	ROM	Not reported	Immediate (Day 0)	Retrospective Case Series	4
Hagiwara et al.	2014	Japan	58.8		56.3	Circumferential + MUA		20.4	Not reported	UCLA, Pain, Function, MMT, ROM	Until pain subsided	Not reported	Retrospective Cohort	3
Hagiwara et al.	2015	Japan	41.7		65.1	Circumferential		6	Not reported	ASES, UCLA, Constant, ROM	Not reported	Not reported	Case Series	4
Hagiwara et al.	2020	Japan	Not reported		Not reported	Circumferential		Not reported	Not reported	ROM	Not reported	After pain subsides	Case Series	4
Harryman et al.	1997	USA	53.3		47	Circumferential + MUA		32	Not reported	Not reported	Not reported	Not reported	Prospective Cohort	2
Hasegawa et al.	2020	Japan	21.7		60.1	Anterior + MUA		13.1	3	VAS, JOA, ASES, ROM 6mo	Not reported	Immediate (Day 1)	Retrospective Cohort	3
Holloway et al.	2001	USA	Not reported		Not reported	Circumferential + MUA		20	6	Postoperative Shoulder Score	Not reported	Immediate (Day 0)	Prospective Cohort	2
Jeong et al.	2020	Korea	37.9		61.37	Circumferential + MUA		32.51	18	VAS, Constant, ASES, ROM 6mo, 1yr	4 weeks (small-med tears) 6 weeks (large-massive tears)	Delayed (4–6 weeks)	Prospective Cohort	3
Jerosch et al.	2001	Germany	46.4		49	Circumferential		22	Not reported	Constant, ROM intraoperative, 6wks, final	Not reported	Immediate (Day 0)	Prospective Cohort	2
Jerosch et al.	2013	Germany	49.1		Not reported	Circumferential		36	Not reported	Constant, VAS	Not reported	Immediate (Day 0)	Retrospective Case Series	4
Kim et al.	2020	Korea	30.0		55.3	Circumferential + MUA		Not reported	Not reported	VAS, ASES, ROM 3mo, 6mo, 12mo	Not reported	Immediate (Day 0)	Comparative Case Control	3
Lafosse et al.	2012	France	30.0		47	Circumferential		42	Not reported	Constant, VAS, ROM 3mo	Not reported	Immediate (Day 0)	Case Series	4
Le Lievre et al.	2012	Australia	44.2		61	Circumferential + MUA		84	22	ROM	Not reported	Immediate (Day 1)	Retrospective Cohort	3
Levy et al.	2008	United Kingdom	57.1		54	Circumferential		33	Not reported	ROM postop, 6mo, final	Not reported	Immediate (Day 0)	Retrospective Review	4
Rizvi et al.	2019	Australia	33.3		55	Circumferential + MUA		Not reported	30	Patient-reported symptoms, ROM	Not reported	Immediate (Day 1)	Retrospective Cohort	3
Schoch et al.	2020	USA	16.5		56.6	Circumferential + MUA		6.2	4	ROM	Not reported	Immediate (24–48 h)	Retrospective Cohort	3
Su et al.	2019	Taiwan	36.2		55.6	Circumferential + MUA		Not reported	Not reported	ROM	Not reported	Immediate (Day 0)	Retrospective Cohort	3
Waszczykowski et al.	2014	Poland	37.0		51.6	Circumferential + MUA		24	Not reported	Function of flexors muscle of the shoulder (FFLX), ROM, ASES, muscular strength	Not reported	Not reported	Retrospective Cohort	3
Yanlei et al.	2019	Singapore	Not reported		55.5	Circumferential		Not reported	9	Isometric strength, VAS, ROM, Constant, Constant Shoulder, UCLA, Oxford	Up to 1 week	Delayed (2 weeks)	Retrospective Review of Prospectively Collected Data	3
Yildiz et al.	2018	Turkey	16.7		47.7	Circumferential		26	Not reported	Constant, VAS, ROM	Not reported	Immediate (Day 0)	Retrospective Cohort	3
Beaufils et al.	1999	France	24.0		48	Anterior + MUA		21	Not reported	Constant, ROM, Subjective Assessment, Constant	Not reported		Retrospective Cohort	3
Cinar et al.	2010	Turkey	7.7		50	Circumferential		54.1	Not reported	ROM	1 day	Immediate (Day 1)	Retrospective Cohort	3
Gerber et al.	2001	Switzerland	82.2		50.8	Circumferential + MUA		26	Not reported	Constant, ROM, Work, Recreation, Sleep, Use of arm in daily living, Strength, work incapacity, Subjective Shoulder value	Not reported	Not reported	Retrospective Cohort	3
Massoud et al.	2002	United Kingdom	Not reported		48	Circumferential + MUA		29.2	6	Constant Score	Not reported	Not reported	Prospective Cohort	2
Mubark et al.	2015	United Kingdom	27.5		48.2	Circumferential + MUA		6		Pain, activity of daily living, ROM, strength, Constant	Not reported	Immediate (Day 0)	Prospective Cohort	2
Ogilvie-Harris et al.	1997	Canada	52.6		37	Anterior		32.4	2	Pain score, ROM score, Function score	Not reported	Not reported	Prospective Cohort	2
Pearsall et al.	1999	USA	51.2		49	Anterior		22	3	ROM	1 day	Immediate (Day 1)	Prospective Cohort	2
Ranalletta et al.	2017	Argentina	31.3		49.6	Anterior + MUA		63	21	Constant Score, VAS, ROM 8weeks, 6mo, final	Within 24 h	Immediate (week of surgery)	Case Series	4
Snow et al.	2009	United Kingdom	18.8	10.4	51	Anterior + MUA (27)	Circumferential + MUA (21)	5	Not reported	Constant, ROM, Satisfaction score	Not reported	Not reported	Retrospective Cohort	3

### Study quality

In this review, the 46 studies include two (4.3%) studies with Level I evidence, 16 (34.8%) Level II evidence, 18 (39.1%) Level III evidence and 10 (21.7%) Level IV evidence (Table 1). Agreement between reviewers was substantial at the title and abstract screening stage (κ = 0.79, 95% CI 0.72–0.85) and excellent at the full text screening stage (κ = 0.91, 95% CI 0.84–0.99). The mean MINORS score for non-randomized comparative studies was 17 (SD 2.2) and for non-comparative studies 12.2 (SD 1.8), indicating fair quality. The RCTs had low risk of bias (Appendix 2). There was excellent inter-rater agreement for quality assessment according to the MINORS criteria (ICC = 0.99, 95% CI 0.98 to 1.0).

### Surgical techniques

The anterior capsule was released in 10 studies,^18–27^ the entire capsule (360°) in 30 studies^7,12,13,28–54^ and a comparison of both techniques was employed in 6 studies.^10,55–59^ Additional procedures primarily include concomitant MUA in 25 studies, 7 of which were performed with anterior release (70%)^18–20,23,25–27^ and 18 with the circumferential release (60%).^28,29–31,35,38–41,43,46–52^ Other procedures used on a case-by-case basis include concomitant subacromial bursectomy or decompression.^13,18,24,27,30,34,42,45,54^ Surgical operations were carried out in the lateral decubitus in 18 studies (39.1%),^12,13,19–21,26,29–31,34,35,41,43,45,47,51,56,58^ beach chair position in 23 studies (50.0%),^10,24,25,27,28,32,33,36–40,44,46,48–50,52–55,57,59^ an unspecified position in five studies (10.9%).^7,18,22,23,42^ Among the studies that carried out anterior capsular release, lateral decubitus was used in four studies (40.0%),^19–21,26^ beach chair in three studies (30.0%),^24,25,27^ and unspecified in three studies (30.0%).^18,22,23^ Among studies using circumferential release, 16 used beach chair (53.3%),^28,32,33,36–40,46,48–50,52–54^ 12 used lateral decubitus (40.0%),^12,13,29–31,34,35,41,43,45,47,51^ two were unspecified (6.7%).^7,42^ Comparative studies used beach chair in 4 studies (66.7%)^10,55,57,59^ and lateral decubitus in 2 studies (33.3%).^56,58^

### Rehabilitation protocol

Rehabilitation was aimed at enhancing recovery, improving ability to perform daily living tasks and allowing for quicker return to activity. Commonly used techniques included mobilization after surgery consisting of passive and active ROM. Rehabilitation programs began on the day of surgery in 15 studies,^7,20,31,33,34,40,42–45,48,51,54,57^ the day after surgery in 16 studies^10,12,13,19,22,24,26–28,30,32,46,49,50,56,58^ and within 1–4 weeks in 4 studies.^23,25,41,53^ Immobilization ranged from 1 day^25^ to 6 weeks.^41^ ROM exercises were implemented as early as immediately post-surgery^7,20,29,31,33,34,40,43–45,48,51,54,57^ and at 4 weeks^41^ at the latest. Each included study's respective rehabilitation protocol is described in Appendix 1.

### Clinical and radiographic outcomes

#### Anterior capsular release

Among studies reporting VAS for pain for patients who underwent anterior capsular release, three studies reported significant (*p* < 0.001) improvements postoperatively at a mean of 13.1 months,^19^ 63 months^25^ and 28.2 months.^26^ There were two studies that reported significant (*p* < 0.001) improvements postoperatively for Constant scores at a mean of 63 months^25^ and 28.2 months.^26^ The reported improvement in postoperative forward flexion was significant (*p* < 0.001) in three studies at a mean of 13.1 months,^19^ 63 months^25^ and 28.2 months^26^ with a pre-operative mean of 89.7° (SD 11.4°) and post-operative mean of 163.8° (SD 8.8°).^19,25,26^ There were four studies that reported significant (*p* ≤ 0.003) improvements postoperatively for abduction from a mean of 78.4° (SD 30.4°) to a mean of 155.1° (SD 12.4°) at final follow-up at a mean of 13.1 months,^19^ 32.4 months^21^ and 63 months^25^ and 28.2 months.^26^ Improvements in internal rotation postoperatively was reported to be significant (*p* < 0.001) in three studies.^19,25,26^ The pre-operative mean was 11.7° (SD 6.1°) and mean at final follow-up was 47.3° (SD 23.5°). In four studies, significant (*p* ≤ 0.004) improvements were reported postoperatively for external rotation with a pre-operative mean of 13.3° (SD 3.9°) to a mean of 53.3° (SD 10.9°) at final follow-up.^19,21,25,26^ A significant improvement (*p* < 0.001) in ASES was observed in two studies.^14,19^

#### Circumferential capsular release

For patients who underwent circumferential capsular release, the reported VAS for pain scores in four studies demonstrated significant (*p* < 0.05) improvements postoperatively.^29,33,42,44^ Significant (*p* < 0.05) improvements postoperatively with respect to Constant scores.^33,35,36,42,44,48^ and ASES^29,31,36^ were reported. The reported improvement in postoperative forward flexion was significant (*p* < 0.03) in 10 studies from a mean pre-operative value of 85.8° (SD 19.4°) to a mean of 157° (SD 15.2°) at final follow-up.^12,13,28,30,33,35,36,44–46^ There were nine studies that reported significant (*p* ≤ 0.05) improvements postoperatively for abduction with a pre-operative value of 63.4° (SD 15.4°) to 151.2° (SD 13.3°) at final follow-up.^13,28–30,33,42,44–46^ Improvements in internal rotation postoperatively were reported to be significant (*p* < 0.05) in seven studies from 21.9° (SD 13.1°) pre-operatively to 59° (SD 3.7°) at final follow-up.^29,33–36,42,46^ In 11 studies, significant (*p* < 0.05) improvements were reported postoperatively for external rotation from a mean of 16.7° (SD 8.5°) to 59.7° (SD 13.0°) at final follow-up.^12,13,28–30,33–35,42,44,45^ Among studies that reported forward elevation, two studies reported significant (*p* < 0.05) improvements postoperatively for forward elevation.^29,34^ The mean forward elevation improved from 91.9° (SD 12.9°) pre-operatively to 156.7° (SD 6.3°) at final follow-up. There were two studies reporting significant (*p* < 0.039) improvements postoperatively in UCLA score.^36,38^ The MCID for UCLA associated with rotator cuff repair is 2.9; hence, it is difficult to ascertain whether these differences are clinically significant for patients being treated for AC.^60^

### Comparative outcomes

**Figure 2.**
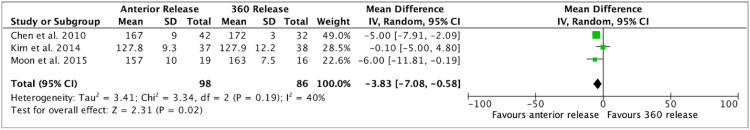
Forward flexion 3 months (degrees).

There were six studies that directly compared anterior and circumferential capsular release.^10,55–59^ The pooled outcomes of two studies reporting forward flexion at 3 months revealed improved ROM for patients undergoing anterior release compared to circumferential release (*P* < 0.02) (MD, −3.83; 95% CI, −7.08 to −0.58, I^2^ = 40%) (Figure 2). Pooled outcomes for other surgical outcome measures including internal rotation and VAS for pain at different postoperative follow-up times yielded non-significant results.

### Complications

The overall complication rate among all patients included in this systematic review was 0.84% (n = 11). Recurrence of AC was reported in nine cases (0.40%) (8 circumferential and 1 anterior capsular release).

In those undergoing circumferential release, the complication rate was 0.44% (n = 8) and complications include superficial infection (n = 2),^25^ recurring pain (0.06%; n = 1),^25^ avascular necrosis (0.06%; n = 1),^45^ postoperative infection (0.06%; n = 1),^42^ delayed healing of the posterior portal (0.06%; n = 1).^29^ Recurrence occurred in eight cases (0.35%). Subsequent release was performed in five patients, and a second operation, surface prosthesis and open rotator cuff repair was performed in the remaining three patients.^42^

In the group of patients that underwent anterior capsular release, the complication rate was 0.67% (n = 3) and complications included superficial infection (0.44%; n = 2)^18–26^ and prolonged postoperative pain (0.22%; n = 1).^25^ Recurrence was reported in one patient (0.04%) which was treated with corticosteroid injections.^25^

## Discussion

The most significant finding of this systematic review was that there were no clinically significant differences found in outcome measures at different post-operative time points between anterior and circumferential capsular release patients. Also of significance is the relatively low complication rate in both the anterior and circumferential release groups (0.67% vs 0.44%). Furthermore, significant postoperative improvements were seen in both groups of patients, particularly in the postoperative outcomes of VAS for pain, Constant scores, and ROM in forward flexion, abduction, internal rotation and external rotation. Forward elevation and UCLA scores were additionally found to be significantly improved for patients who underwent circumferential capsular release however these differences are unlikely to be clinically significant given they did not reach the MCID for the UCLA.^60^ The pooled analysis of studies directly comparing anterior capsular release with 360° release revealed no significant differences between the two surgical techniques at all postoperative time periods (e.g. 3 months, 6 months, 12 months, final follow-up). Overall, both procedures provide clinically similar outcomes with respect to function and ROM. Rates of recurrence were also low for both anterior and circumferential techniques (0.04% vs 0.35%).

In the current systematic review, the majority of patients underwent the circumferential capsular release (80.1%). The increased prevalence of circumferential release may potentially be related to the concept of posterior capsular release improving glenohumeral internal rotation which is restricted by the posterosuperior capsule.^58,61,62^ It has been reported that circumferential may result in faster recovery, however in a 2015 RCT, there was no significant difference in outcomes between the two techniques at final follow-up.^56^ Employing the circumferential release technique also may avoid the need for manipulation in the majority of cases.^12^ In this systematic review, the rate of MUA in the anterior release group was higher than the circumferential release group. MUA is routinely carried out during capsular release, and presents a number of possible complications.^12^ These include increased risk of dislocation, humeral fracture, rotator cuff tear, brachial plexus injury, joint hemorrhage and inadvertent soft-tissue injury.^12,26,44,57^

The low complication rates in both the anterior release technique and circumferential release technique are notable. Controlled capsular release as opposed to isolated MUA decreases the risk of injury to the soft tissue structures and bone and likely results in earlier recovery and improved rehabilitation.^12,44^ Although the choice for surgical positioning will largely depend on the surgeon's preferences, there are benefits to both the lateral decubitus and beach chair position. While the lateral decubitus is said to offer improved visualization of the shoulder capsule, the beach chair position allows for intraoperative movement of the humeral head with ease.” Recent published results encourage a transition from the more commonly used beach chair position to a lateral decubitus approach due to improved visualization, and potentially overall reduced risk of iatrogenic injury and low complication and revision rates.^11,12^ The improved visualization, particularly in the inferior capsule and axillary recess, further eliminates the need for manipulation.^11^ The findings of this systematic review do not report differences in patient positioning. Future studies should aim to assess outcomes of anterior release and 360° release based on surgical positioning to determine an optimal treatment method.

Several factors contribute to the post-operative success of arthroscopic capsular release including the timing of surgical intervention and etiology.^49^ A recent systematic review found that despite a comparable success rate, diabetic patients experience increased recurrent pain post-surgically and poorer ROM and function compared to idiopathic cases.^63^ The arthroscopic capsular release procedure, however, was shown to be equally effective in patient groups classified by etiology including postsurgical, idiopathic, post-traumatic, and diabetic.^53,64^ The surgical technique selected for each patient should be dictated by the restriction in ROM exhibited by the patient.^59^ The superior and middle glenohumeral ligaments, the rotator interval, the coracohumeral ligament extra-articularly and/or the intra-articular portion of the subscapularis should be released to address loss of external rotation while release of the anteroinferior capsule addresses loss of elevation.^59^ A posterosuperior release can be performed to address loss of internal rotation.^59^

### Limitations

Some studies had poor documentation of patient demographics and surgical techniques and had to be excluded from the review. Furthermore, the pooling of outcomes was limited due to the lack of available studies for this purpose. Pooling across multiple studies was not possible due to the large volume of studies included, each reporting different outcome measures. There was a lack of detail provided regarding how MUA was performed between the study groups, limiting our ability to determine its effect on postoperative outcomes and complications. There were also several inconsistencies in complications reporting as some studies did not report complications at all, whereas others reported only major complications leading to revision while excluding minor complications. Thus, the overall complication rates found in this systematic review may be subject to inaccuracies and be possibly underestimated/underreported. Although statistically significant differences were found they were unlikely to be clinically significant. The included studies had varying follow-up periods; thus, it was difficult to ascertain the trajectory of improvement postoperatively. The variability between studies in postsurgical treatment approach, including physiotherapy technique and prescribed analgesic, is notable. As these choices may directly influence the final outcome of the surgical intervention, making direct comparisons was not possible. A lack of documentation in outcomes distinguishing between primary AC and secondary AC patients limited our ability to determine the influence of etiology on outcomes. Thus, there was a limited ability to make adequate comparisons between the two techniques. Additionally limited high quality studies are available to inform treatment decisions.

### Future studies

Future studies should aim to improve documentation of patient demographics, etiology of AC, surgical techniques, rehabilitation protocol and outcomes. The effect of time of onset of symptoms, as well as delay in treatment on patient outcomes should be assessed. This will allow clinicians to determine the ideal time to treat patients based on clinical symptoms and patient history. A comparison between patient positioning and surgical techniques will be needed to determine an optimal treatment method. Future studies should specify the stage of AC to assess outcomes for similar patient groups. Furthermore, the class of stiffness must be defined to ensure consistent conditions in comparing procedures. This can be accomplished using an RCT study design with large sample sizes in order to limit inclusion bias. In addition, a guideline for clinicians considering patient history, imaging and surgical findings should be established to determine an appropriate treatment for patients. The development and implementation of a standardized MUA protocol is needed for research purposes with the intent of comparing outcomes of different surgical techniques. Furthermore, as the current literature reports distinct combinations of outcome measures, a set of standardized core outcomes for AC should be instituted. The differences in postsurgical treatment method likely influenced the outcomes and should be taken into account. Hence, RCTs with a clearly defined physiotherapy and pain protocol are needed to rule out bias associated with post-surgical rehabilitation. Complete reporting of outcomes and complications will permit for a more in-depth analysis and comparison between anterior release and circumferential release.

## Conclusion

Anterior capsular release and circumferential capsular release both result in significant improvements for patients with AC with respect to ROM as well as functional outcomes with very low complication rates with both procedures. The most significant finding of this systematic review was that there were no clinically significant differences found in outcome measures at different post-operative time points between anterior and circumferential capsular release patients.

## Supplemental Material

sj-docx-1-sel-10.1177_17585732221092016 - Supplemental material for Comparing surgical outcomes of anterior capsular release vs circumferential release for persistent capsular stiffness Click here for additional data file. Supplemental material, sj-docx-1-sel-10.1177_17585732221092016 for Comparing surgical outcomes of anterior capsular release vs circumferential release for persistent capsular stiffness by Safiya Erdogan, Seaher Sakha, Ajaykumar Shanmugaraj, Carlos Prada, Rachel M Frank, Timothy Leroux and Moin Khan in Shoulder & Elbow

sj-docx-2-sel-10.1177_17585732221092016 - Supplemental material for Comparing surgical outcomes of anterior capsular release vs circumferential release for persistent capsular stiffness Click here for additional data file. Supplemental material, sj-docx-2-sel-10.1177_17585732221092016 for Comparing surgical outcomes of anterior capsular release vs circumferential release for persistent capsular stiffness by Safiya Erdogan, Seaher Sakha, Ajaykumar Shanmugaraj, Carlos Prada, Rachel M Frank, Timothy Leroux and Moin Khan in Shoulder & Elbow

sj-docx-3-sel-10.1177_17585732221092016 - Supplemental material for Comparing surgical outcomes of anterior capsular release vs circumferential release for persistent capsular stiffness Click here for additional data file. Supplemental material, sj-docx-3-sel-10.1177_17585732221092016 for Comparing surgical outcomes of anterior capsular release vs circumferential release for persistent capsular stiffness by Safiya Erdogan, Seaher Sakha, Ajaykumar Shanmugaraj, Carlos Prada, Rachel M Frank, Timothy Leroux and Moin Khan in Shoulder & Elbow

## Abbreviations

AC: Adhesive Capsulitis
ASES: American Shoulder and Elbow Surgeons
CI: Confidence Intervals
ICC: Intraclass Correlation Coefficient
JBJS: Journal of Bone and Joint Surgery
MCID: Minimally Clinically Important Difference
MD: Mean Difference
MINORS: Methodological Index for Non-randomized Studies
MUA: Manipulation Under Anesthesia
OR: Odds Ratio
PRISMA: Preferred Reporting Items for Systematic Review and Meta-Analyses
RCT: Randomized Control Trial
ROM: Range of Motion
SD: Standard Deviation
UCLA: University of California at Los Angeles
VAS: Visual Analog Scale.
