# Supplementary material for: Comparing surgical outcomes of anterior capsular release vs circumferential release for persistent capsular stiffness
Source: Shoulder Elbow. 2022 Apr 5;15(4):360–72. doi: 10.1177/17585732221092016 (PMC10395412; doi:10.1177/17585732221092016)
Supplement: sj-docx-1-sel-10.1177_17585732221092016 - Supplemental material for Comparing surgical outcomes of anterior capsular release vs circumferential release for persistent capsular stiffness [file sj-docx-1-sel-10.1177_17585732221092016.docx]

**APPENDIX I Search Strategy**

| **MEDLINE** 282 results | **EMBASE** 677 results | **Cochrane** 83 results | **PubMed** 51 results |
| --- | --- | --- | --- |
| 1. Adhesive capsulitis*.mp 2. Frozen shoulder.mp 3. 1 or 2 4. Adhesive capsulitis/su 5. Release/mp 6. Arthroscop*.mp 7. Anterior capsular release.mp 8. Selective capsular release.mp 9. 360degree capsular release 10. 4 or 5 or 6 or 7 or 8 or 9 11. 3 and 10 12. Limit 11 to (English language and humans) | 1. Adhesive capsulitis*.mp 2. Frozen shoulder.mp 3. 1 or 2 4. Adhesive capsulitis/su 5. Release/mp 6. Arthroscop*.mp 7. Anterior capsular release.mp 8. Selective capsular release.mp 9. 360degree capsular release 10. 4 or 5 or 6 or 7 or 8 or 9 11. 3 and 10 12. Limit 11 to (English language and humans) | 1. (adhesive capsulitis*);ti,ab,kw 2. (frozen shoulder);ti,ab,kw 3. {OR #1-#2} 4. (pancapsular release);ti,ab,kw 5. (release);ti,ab,kw 6. (arthroscop*);ti,ab,kw 7. (anterior capsular release);ti,ab,kw 8. (selective capsular release);ti,ab,kw 9. (360degree capsular release);ti,ab,kw 10. {OR #4-#9} 11. #3 and #10 12. Limit to human and English language | **Search: (((adhesive capsulitis*) OR (frozen shoulder)) AND (((((((adhesive capsulitis/su) OR (pancapsular release)) OR (release)) OR (arthroscop*)) OR (anterior capsular release)) OR (selective capsular release)) OR (360degree capsular release))) AND (("2019/10/06"[Date - Publication] : "2020/10/06"[Date - Publication]))** |
