# Supplementary figures and images for: Comparing surgical outcomes of anterior capsular release vs circumferential release for persistent capsular stiffness
Source: Shoulder Elbow. 2022 Apr 5;15(4):360–72. doi: 10.1177/17585732221092016 (PMC10395412; doi:10.1177/17585732221092016)

**APPENDIX II Cochrane Risk of Bias Summary**


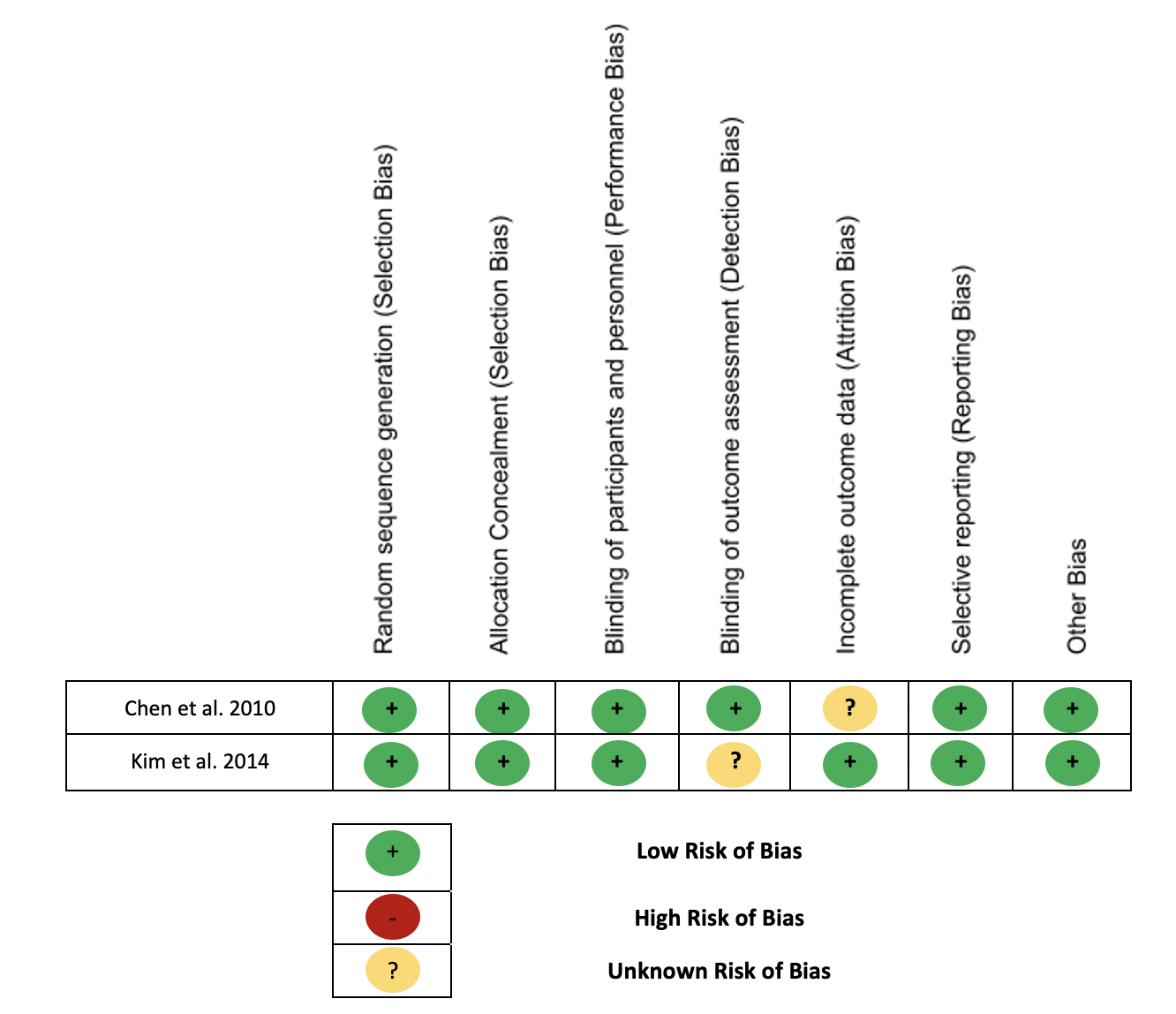

Supplement: sj-docx-2-sel-10.1177_17585732221092016 - Supplemental material for Comparing surgical outcomes of anterior capsular release vs circumferential release for persistent capsular stiffness [file sj-docx-2-sel-10.1177_17585732221092016.docx]
