# Supplementary material for: Comparing surgical outcomes of anterior capsular release vs circumferential release for persistent capsular stiffness
Source: Shoulder Elbow. 2022 Apr 5;15(4):360–72. doi: 10.1177/17585732221092016 (PMC10395412; doi:10.1177/17585732221092016)
Supplement: sj-docx-3-sel-10.1177_17585732221092016 - Supplemental material for Comparing surgical outcomes of anterior capsular release vs circumferential release for persistent capsular stiffness [file sj-docx-3-sel-10.1177_17585732221092016.docx]

**APPENDIX III Rehabilitation Protocol**

| Primary Author, Year | Immobilization (# weeks) | Passive/active (start date) | Physical Therapy | Strengthening | Other |
| --- | --- | --- | --- | --- | --- |
| Bennett W.F., 2000 |  |  | Outpatient during week 1 post-surgery |  |  |
| Chen J., 2010 | 4 | Day 1 | ROM exercises limited to 90° overhead; all directions starting week 4 |  |  |
| Kim Y., 2014 |  | Day 1 | ROM, pulley exercises | Theraband, dumbbells after 1 month |  |
| Moon N.M., 2015 |  | Day 1 | Joint exercises with rod+pulley, active exercises from week 6 | After 3 months |  |
| Oglivie-Harris D.J., 1995 |  | Immediately post-release | Active assisted ROM hourly, physical therapy 3x/week for 6 weeks |  |  |
| Ozaki J., 1989 |  | Day 3 | Active mobilization, pendulum exercises on day 3, encouraged to use arm in daily activities at week 3 |  |  |
| Tsai M.J., 2017 |  | Day 1 | Passive stretching 15 min/day for 6 weeks |  | Sling with small pillow in slight abduction |
| Warner J.J., 1996 |  | Morning Day 1 | Morning and afternoon sessions for 2 days, daily sessions and self-assisted motion exercise program for 2 weeks, followed by 4 weeks of 2-3 PT sessions then individualized programming |  |  |
| Omari A., 2001 |  | Morning Day 1 | ROM exercises |  |  |
| Barnes C.P., 2016 |  | Day 1 | Active and passive ROM exercises every 2 hours for remainder of week |  |  |
| Baums M.H., 2007 |  | Immediately post-release | Passive ROM emphasizing forward elevation, external and internal rotation; continuous passive motion 2hrs 2x/day, PT sessions 2x/week |  | Home exercises for 6 weeks post-op |
| Cho C.H., 2016 |  | Immediately post-release (Day 0) | Pendulum and continuous passive-motion exercises, active and passive ROM exercises after discharge |  | Sling |
| Cvetanovich G.L., 2018 |  | Day 1 | ROM with PT 3-4x/week, continuous passive motion devices 4-6hrs/day 4 weeks with forward flexion from 0-90° with advancement by 5° every 30min as tolerated |  | Sling, one-time interscalene block for pain control, |
| Dattani R., 2013 |  | Day 1 |  |  |  |
| De Carli A., 2011 | 2 weeks max | Day 1 | Passive exercises | Beginning week 5 |  |
| Diwan D.B., 2005 |  | Day 0 | Elbow ROM exercises, neck stretches day 0-7, pendular rotations and shoulder ROM after week 1, optional therapist involvement | Grip strengthening with tennis ball, Theraband with rowing and active resistance external rotation 2x/day at 6 weeks | Cryotherapy 20min/hour for first 48hrs and after exercise sessions |
| Ebrahimzadeh M.H., 2014 |  | Immediately post-release | Passive ROM exercises |  |  |
| Fernandes M.R., 2013 |  | Immediately post-release | Immediate PT for 72 hours with passive ROM 2x daily |  | Bolus of 15-20mL 0.5% bupivacaine 30minutes before each rehabilitation session; |
| Hagiwara Y., 2014 | Until pain subsided |  | Initiated during sling immobilization |  |  |
| Hagiwara Y., 2020 |  | After pain subsides |  |  | Sling worn for pain management for a few days |
| Harryman D.T., 1997 |  |  | No formal physical therapy program |  | Stretches for 5 minutes 5x/day |
| Hasegawa A., 2020 |  | Day 1 | Passive ROM 2-3x/day, PT program 2-3 days/week after discharge 7-14 days postop |  |  |
| Holloway G.B., |  | Day 0 |  | Theraband exercises introduced when pain disappeared | Exercise program on day of surgery consisting of 5 exercises for 2min done 6x/day |
| Jeong J.Y., 2020 | 4 weeks (small-medium tears)  6 weeks (large-massive tears) | At 4 weeks | Passive ROM exercises, active assisted ROM once full passive ROM recovered | Started between 10-12 weeks postop and continued 3-6 months | Immobilization using 30 abduction pillow (4 weeks recommended for small-medium size tears and 6 weeks for large or massive tears) |
| Jerosch J., 2001 |  | Day 0 | Active ROM, 2x/week supervised PT sessions with home-exercise program | Strengthening shoulder muscles once active shoulder and postop pain subsided | 5-7 day admission to hospital postop |
| Jerosch J., 2013 |  | Day 0 | Daily PT session with exercises, daily therapy on continuous passive motion machine 6 weeks |  | IV pain-control therapy consisting 2 ampoules Tramal and Novalgin and ampoule of Gastrosil in early postop period |
| Kim D.H., 2020 |  | Day 0 | Pendulum exercises, immediate passive ROM exercises |  |  |
| Lafosse L., 2012 |  | Day 0 | PT with full passive and active ROM as tolerated |  | Oral analgesia |
| Le Lievre H.M., 2012 |  | Day 1 | ROM exercises, 2 PT sessions for passive and active- assisted shoulder motion, assisted shoulder movements every 2hours at home for remainder of week, 10 reps active-assisted external rotation of shoulder with broom handle  Week 2: 5 reps 10sec stretches for flexion, horizontal adduction, external rotation (0° and 90°) | Week 2: 3 sets of 10 reps daily resistive retraction with Theraband for external rotation, internal rotation and adduction. Free weight resistive flexion and abduction  Rotator cuff muscle exercises at home 3x/day for 10 weeks | Ice pack for 20 minutes every 2 hours during waking hours for 2 days |
| Levy O., 2008 |  | Day 0 | Starting in recovery unit, pendulum movement, passive assisted exercise, stretching | Rotator cuff strengthening at later stages |  |
| Rizvi S.M., 2019 |  | Day 1 | Meet with PT minimum 2x/week for 6 weeks for active/passive motion, advised to perform pendular reach, shoulder flexion and shoulder extension 3x/day, shoulder stretch 3x/day | Day 6: Begin rotator cuff exercises with Theraband 3x/day for 12 weeks |  |
| Schoch B., 2020 |  | Initiated 24-48hrs post-release | 2-3/week with home-based exercises, passive and active stretching, followed-up at clinic in 2 weeks and returned after 5 weeks (2 weeks if progressing slowly | Passive and active strengthening | Discharged with soft sling in place |
| Su Y.D., 2019 |  | Day 0 | Pendulum exercise and passive ROM, home exercise program with follow-up at orthopedic clinic |  | Sling protection; cryotherapy |
| Yanlei G.L., 2019 | Up to 1 week | 2 weeks post-release | Passive ROM, active ROM when pain well-controlled |  | Arm sling 1 week |
| Yildiz F., 2018 |  | Day 0 | Forward flexion, abduction, external and internal rotation, active ROM allowed as patient tolerated, daily PT continued minimum 1 month after discharge |  | Interscalene blockage continued through catheter for pain control |
| Cinar M., 2010 | 1 day | Day 1 | Passive and active motions started after sling removal |  |  |
| Gerber C., 2001 |  |  | Passive PT on inpatient basis 2-4 days, PT sessions 3x/week to gain passive ROM with emphasis on anterior elevation |  | Interscalene catheter for 2-4 days |
| Massoud S.N., 2002 |  |  | Daily outpatient PT for first few days, home stretching exercise program |  |  |
| Mubark I.M., 2015 |  | Day 0 | Pendulum exercises, stretching against door jam and butterfly motions, home-based shoulder stretching programme 5-10min performed 4-5x/day with 6 exercises in forward elevation, external rotation, internal rotation and cross body adduction |  |  |
| Pearsall A.W., 1999 | 1 day | Day 1 | Passive ROM, progressive passive and active-assisted ROM exercises including aquatics starting week after surgery |  | Narcotics for pain, 21-day tapered course for prednisone; cryotherapy |
| Ranalletta M., 2017 | Within 24 hours | The week of surgery | 3x PT visists/week until end of treatment |  | Encouraged to discontinue use of sling 24hrs postop and start using operated arm for activities of daily living; |
